# Application of machine learning algorithms to construct and validate a prediction model for coronary heart disease risk in patients with periodontitis: a population-based study

**DOI:** 10.3389/fcvm.2023.1296405

**Published:** 2023-11-29

**Authors:** Yicheng Wang, Binghang Ni, Yuan Xiao, Yichang Lin, Yu Jiang, Yan Zhang

**Affiliations:** ^1^ Department of Cardiovascular Medicine, Affiliated Fuzhou First Hospital of Fujian Medical University, Fuzhou, Fujian, China; ^2^ The Third Clinical Medical College, Fujian Medical University, Fuzhou, Fujian, China; ^3^ Cardiovascular Disease Research Institute of Fuzhou City, Fuzhou, Fujian, China; ^4^ The Graduate School of Fujian Medical University, Fuzhou, Fujian, China

**Keywords:** periodontitis, coronary heart disease, machine learning, prediction model, NHANES, national health and nutrition examination survey

## Abstract

**Background:**

The association between periodontitis and cardiovascular disease is increasingly recognized. In this research, a prediction model utilizing machine learning (ML) was created and verified to evaluate the likelihood of coronary heart disease in individuals affected by periodontitis.

**Methods:**

We conducted a comprehensive analysis of data obtained from the National Health and Nutrition Examination Survey (NHANES) database, encompassing the period between 2009 and 2014.This dataset comprised detailed information on a total of 3,245 individuals who had received a confirmed diagnosis of periodontitis. Subsequently, the dataset was randomly partitioned into a training set and a validation set at a ratio of 6:4. As part of this study, we conducted weighted logistic regression analyses, both univariate and multivariate, to identify risk factors that are independent predictors for coronary heart disease in individuals who have periodontitis. Five different machine learning algorithms, namely Logistic Regression (LR), Gradient Boosting Machine (GBM), Support Vector Machine (SVM), K-Nearest Neighbor (KNN), and Classification and Regression Tree (CART), were utilized to develop the model on the training set. The evaluation of the prediction models’ performance was conducted on both the training set and validation set, utilizing metrics including AUC (Area under the receiver operating characteristic curve), Brier score, calibration plot, and decision curve analysis (DCA). Additionally, a graphical representation called a nomogram was created using logistic regression to visually depict the predictive model.

**Results:**

The factors that were found to independently contribute to the risk, as determined by both univariate and multivariate logistic regression analyses, encompassed age, race, presence of myocardial infarction, chest pain status, utilization of lipid-lowering medications, levels of serum uric acid and serum creatinine. Among the five evaluated machine learning models, the KNN model exhibited exceptional accuracy, achieving an AUC value of 0.977. The calibration plot and brier score illustrated the model's ability to accurately estimate probabilities. Furthermore, the model's clinical applicability was confirmed by DCA.

**Conclusion:**

Our research showcases the effectiveness of machine learning algorithms in forecasting the likelihood of coronary heart disease in individuals with periodontitis, thereby aiding healthcare professionals in tailoring treatment plans and making well-informed clinical decisions.

## Introduction

Periodontitis is an inflammatory response that affecting the periodontal tissues, leading to progressive degradation of the tooth-supporting structures. This pathological process is initiated by a consortium of pathogenic microorganisms presenting within dental plaque (1, 2). As a highly prevalent chronic condition among the global adult population, periodontitis impacts more than 50% of individuals worldwide (3). According to statistics, periodontitis affects many American individuals over the age of 30 (4). However, there has been a recent surge in the prevalence of periodontitis among younger individuals (5).

In the United States and throughout the world, coronary heart disease (CHD) is a chronic cardiovascular illness that exhibits significant morbidity and mortality rates (6, 7). The underlying pathophysiology is that the narrowing or blockage of coronary arteries due to atherosclerosis leads to decreased blood and oxygen supply to myocardial tissues, ultimately resulting in tissue necrosis and the development of the disease (8). Any age can get coronary heart disease, and as people get older, their chances of getting it increase (9). Therefore, the recognition and control of traditional risk elements associated with CHD, including smoking, alcohol intake, high blood pressure, obesity, diabetes mellitus, and lack of physical activity, are pivotal in both preventing and managing CHD (10).

Prior research has firmly established a strong association between the development of cardiovascular diseases, such as CHD, and the presence of elevated inflammation levels (6, 11). Multiple research studies have provided evidence indicating a correlation between periodontitis and an elevated likelihood of developing cardiovascular disease (12–15). Periodontitis also has an impact on the outcome of individuals with cardiovascular disease and raises the likelihood of mortality (16). This association may be explained by inflammatory mediators since periodontitis can alter the amounts of inflammatory markers in the blood (17). The bacteremia and systemic inflammatory state associated with periodontitis plays a significant role in the development of vascular endothelial lesions and the enhancement of inflammatory processes in the vascular wall (18). Through the induction of a systemic inflammatory response and autoimmune illness, a chronic infection brought on by periodontitis progresses to atherosclerosis and, ultimately, coronary heart disease (19).

In recent years, machine learning has become a powerful computerized method for analyzing data and has been widely embraced in the field of medicine as an effective tool for predicting disease risks (20–25). Several studies have shown that clinical prediction models utilize the powerful predictive power of machine learning algorithms to outperform traditional statistical methods (26, 27). Considering the increasing association between periodontitis and cardiovascular diseases, including CHD, no studies have been reported on prediction model for CHD risk in patients with periodontitis. Therefore, a prediction model that integrates risk factors is needed to assess the risk of CHD in patients with periodontitis. Our study utilizes data from NHANES spanning 2009–2014, in order to construct a prediction model for assessing the risk of CHD among individuals with periodontitis. By employing machine learning algorithms and comparing their performance, our aim is to facilitate personalized clinical decision-making for healthcare professionals. The identification of high-risk patient groups will enable targeted interventions, thereby reducing hospitalization rates and enhancing overall clinical outcomes.

## Methods

### Study design and participants

**Figure 1:**
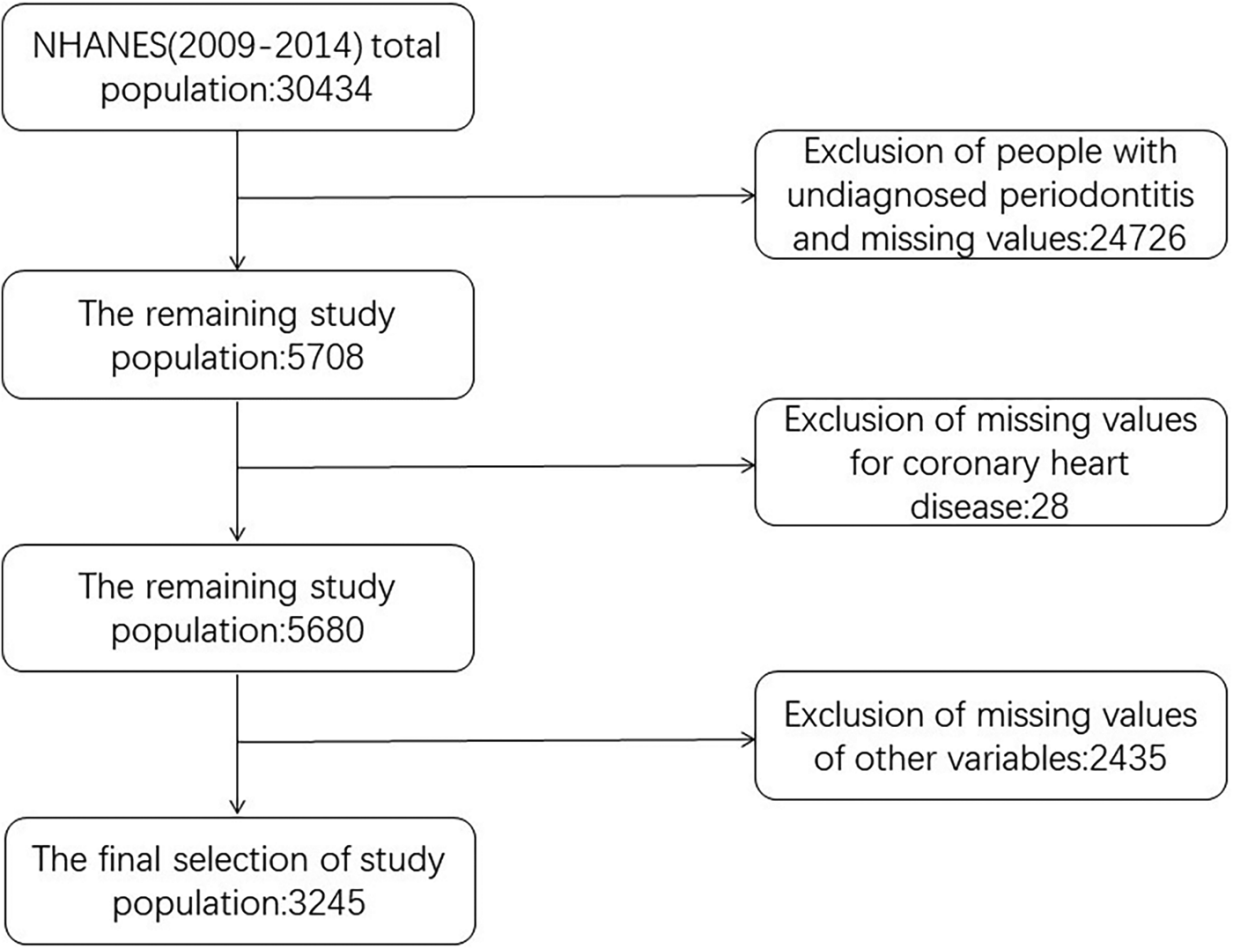
ROC curve analysis of 5 ML algorithms in the validating set.

NHANES is a research initiative that aims to conduct a comprehensive assessment of the health and nutritional well-being of individuals, including both adults and children, who are living within the borders of the United States. Annually, a representative sample of approximately 5,000 individuals is selected, ensuring national coverage, while the database undergoes updates every two years. The study evaluated the intricate health status of Americans by employing a series of sophisticated stratified, multi-stage sampling designs. Our study used data from a total of 30,434 subjects from three consecutive cycles of NHANES 2009–2014. The exclusion criteria were: (1) Age less than or less than 30 years. (2) Those who were not diagnosed with periodontitis. (3) Missing values for at least one of all variables included in the participants of this study. After inclusion and exclusion, a total of 3,245 subjects aged 30 years and above who participated in a demographic survey, physical examination, and questionnaire with a diagnosis of periodontitis were finally included in our analyses. All individuals involved in the study provided their consent after signing an informed consent document, and the survey protocol received approval from the Research Ethics Review Board at the National Center for Health Statistics. All procedures were carried out following applicable guidelines and regulations. The screening flowchart of the study population is shown in Figure 1.

### Ethics of approval statement

The authors assume complete accountability for all aspects of the research, ensuring thorough investigation and resolution of any inquiries regarding the accuracy or integrity of any segment of the study. The study adhered to the revised 2013 Declaration of Helsinki. Since all data from the NHANES program is publicly accessible and free, there was no necessity for medical ethics committee board approval. Prior to participation, written informed consent was acquired from all participants.

### The definition of periodontitis

Comprehensive periodontal examinations were conducted by a dental hygienist to evaluate the periodontal status of participants. Participants aged 30 years and above were eligible for inclusion in the periodontal assessment if they possessed at least one tooth (excluding the third molar) and did not meet any of the health exclusion criteria. According to the AAP/CDC criteria, periodontitis was assessed and classified into non-existent, mild, moderate, and severe categories based on its severity (28). The total number of periodontitis cases was calculated by aggregating the incidences of mild, moderate, and severe cases.

### The definition of coronary heart disease

The assessment of CHD status primarily relied on questionnaires, wherein participants were asked if a healthcare professional had ever diagnosed them with CHD. Affirmative responses indicated the presence of CHD in the subject.

### Other data selection and measurements

The demographic characteristics can be categorized according to sex (female, male), race (non-Hispanic white individuals, non-Hispanic black individuals, Mexican American, Hispanic American, other races), marital status [unmarried, married or cohabiting with a partner, married but currently living alone (separated, divorced, or widowed)], and educational attainment level (below 9th grade, 9th-11th grade, high school graduate, partial college or AA graduate or higher).

To compute the poverty-to-income ratio (PIR), household (or individual) income was divided by state-specific poverty thresholds corresponding to the survey year. Waist circumference (WC) and body mass index (BMI) were measured by medical professionals at mobile screening stations, with BMI calculated as weight in kilograms divided by height in meters squared (kg/m^2^). Sleep duration on workdays, sedentary behavior, and weekly physical activity were self-reported through a questionnaire. Participants were asked about their daily sitting time to assess sedentary behavior. Sleep duration on workdays was determined by querying participants about their typical amount of sleep received during such days. Physical activity time was obtained by asking subjects about the duration of exercise performed per week.

Smoking status of each participant was assessed through self-report and categorized into three groups: non-smokers, former smokers who are no longer smoking, and current smokers. Drinking status was classified as follows: never drinkers, abstainers after previous drinking, heavy drinkers (≥3 drinks per day for women/≥4 drinks per day for men/five or more days of binge drinking per month), moderate drinkers (≥2 drinks per day for women/≥3 drinks per day for men/≥2 days of binge drinking per puff), and light drinkers (excluding the above). The ACC/AHA proposes a two-stage classification system for blood pressure, wherein hypertension is defined as having a systolic blood pressure (SBP) exceeding 130 mmHg and/or diastolic blood pressure (DBP) below 80 mmHg (29). The question “Have you ever received a diagnosis of high blood pressure, also known as hypertension, from a medical professional?” was employed to ascertain the presence of hypertension in the study participants. Diabetes conditions are categorized into three groups: no diabetes, prediabetes, and diabetes. To assess prediabetes, participants were asked the following question: ‘Have you ever received a diagnosis of pre-diabetes, impaired fasting glucose, impaired glucose tolerance, borderline diabetes or been informed by your doctor or healthcare provider that your blood glucose levels are higher than normal but not high enough to be classified as diabetes mellitus or glucose diabetes?’ Participants who responded affirmatively were considered prediabetic. Similarly, individuals diagnosed with diabetes were identified by asking the question: ‘Have you ever been told by a doctor or health professional that you have diabetes?'“ The use of glucose-lowering drugs can be divided into three types: no use of antihyperglycemic/ antihypertensive/ lipid-lowering, use of antihyperglycemic/ antihypertensive/ lipid-lowering, taking medications prescribed other than antihyperglycemic/ antihypertensive/ lipid-lowering. Various blood biochemical markers such as total cholesterol (TCHOL), albumin (ALB), high density lipoprotein (HDL), uric acid (UA), triglycerides (TG), creatinine (CR), blood urea nitrogen (BUN), and HbA1c are measured from the laboratory.

### Development and validation of five machine learning prediction models

The data was randomly partitioned into training set and validation set in a 6:4 ratio. Five machine learning algorithms, namely Gradient Boosting Machine (GBM), Support Vector Machine (SVM), Logistic Regression (LR), Classification and Regression Tree (CART), and K-Nearest Neighbor (KNN), were employed to construct models on the training set. The performance of each model was assessed on the validation set and compared accordingly. The optimal choice of the machine algorithm is determined by selecting the model with the highest AUC.

The predictive performance of the five machine learning models was assessed by plotting the receiver operating characteristic curve (ROC) in both the training set and validation set, and calculating the AUC. The model with the highest AUC was selected as the optimal choice for each algorithm. Calibration curve analysis and Brier score were employed to examine the correlation between actual probabilities and predicted probabilities for each model. Additionally, DCA was utilized to evaluate the clinical applicability of these five machine learning algorithms.

### Statistical analysis

NHANES employs a stratified multistage probability sampling technique, wherein specific sampling weights are assigned to each participant based on the primary sampling unit, thereby facilitating the generation of nationally representative estimates (30). Following the NHANES analysis guidelines, new sample weights were generated by combining continuous data from three two-year cycles (original two-year sample weights divided by 2). NHANES sample weights were utilized for baseline descriptions and logistic regression analyses. In the baseline information, means and standard errors were used to represent continuous variables, whereas categorical variables were presented as percentages (%) and numbers (*n*). Between-group disparities were assessed by employing t-tests for continuous variables and Fisher's exact tests or chi-square tests for categorical variables. Univariate and multivariate logistic regression models were employed to identify independent risk factors for CHD, with odds ratio (OR) and corresponding 95% confidence intervals (CI) used as effect estimates. Statistical analyses were conducted using R software (version 4.3.0) and SPSS version 28.0, considering *P* < 0.05 as statistically significant.

## Results

### The baseline characteristics of study population

The study included a total of 3,245 participants aged 30 years and older, who had previously been diagnosed with periodontitis, based on data extracted from the NHANES database spanning from 2009 to 2014. The mean age was 54.13 years and included 2,016 males and 1,229 females. There were 41.33% non-Hispanic white persons, 23.11% non-Hispanic black persons, 15.93% Mexican Americans, 9.18% Hispanic Americans, and 10.45% Americans of other races. Regarding the marital status of the participants, 10.63% were categorized as unmarried, while 63.05% were identified as married or cohabiting, and 26.32% reported being divorced or separated. The study population of 3,245 was partitioned into training set and validation set in a ratio of 6:4. The study population was stratified into two groups based on the presence or absence of CHD in both the training set and validation set. In the training set, there were significant statistical differences observed between the two groups with respect to age, race, marital status, smoking and alcohol consumption, waist circumference, uric acid, creatinine, urea nitrogen, albumin, total cholesterol, high-density lipoprotein, HbA1c, time spent in physical activity, myocardial infarction, chest pain, diabetes mellitus, hyperlipidemia, and use of antihyperglycemic/antihypertensive/lipid-lowering medication (*P* < 0.05). In the validation set, there was a notable dissimilarity observed between the two groups concerning age, gender, race, uric acid, creatinine, urea nitrogen, smoking status, hypertension status, myocardial infarction status, chest pain status, diabetes mellitus status, and the use of antihyperglycemic/antihypertensive/lipid-lowering medication(*P* < 0.05). The demographic profile of the study sample is presented in Table 1.

**Table 1:** Weighted baseline characteristics.

Variables	Total	Training	Set	*P*-value	Validating	Set	*P*-value
		No	Yes		No	Yes	
*N*	3,245	1,884	63		1,252	46	
AGE (years)	54.13 (0.36)	53.56 (0.42)	67.78 (1.54)	<0.001	53.86 (0.54)	65.22 (2.75)	<0.001
BMI (kg/m^2^)	28.84 (0.16)	28.64 (0.20)	30.19 (1.01)	0.13	29.05 (0.21)	29.72 (1.27)	0.61
WC (cm)	100.52 (0.37)	99.99 (0.46)	108.23 (2.62)	0.003	100.77 (0.57)	104.96 (3.28)	0.23
PIR	2.92 (0.06)	2.91 (0.06)	2.89 (0.24)	0.92	2.91 (0.07)	3.27 (0.29)	0.2
SLEEP (hours)	6.81 (0.03)	6.81 (0.04)	6.88 (0.19)	0.71	6.80 (0.04)	6.85 (0.22)	0.85
Sedentary (min)	349.43 (5.33)	349.62 (5.57)	358.70 (24.29)	0.72	346.67 (7.57)	396.66 (35.90)	0.15
Exercise (min)	646.57 (19.89)	636.00 (23.17)	420.14 (104.21)	0.04	679.87 (28.47)	540.11 (165.96)	0.39
UA (mg/dl)	5.57 (0.04)	5.56 (0.05)	6.28 (0.28)	0.02	5.53 (0.05)	6.14 (0.28)	0.04
CR (mg/dl)	0.91 (0.01)	0.91 (0.01)	1.08 (0.07)	0.02	0.90 (0.01)	1.09 (0.04)	<0.001
BUN (mg/dl)	13.71 (0.14)	13.57 (0.16)	16.61 (0.61)	<0.001	13.68 (0.20)	15.71 (0.81)	0.01
ALB (mg/dl)	4.27 (0.01)	4.27 (0.01)	4.09 (0.05)	0.002	4.27 (0.01)	4.31 (0.05)	0.53
TG (mg/dl)	163.92 (3.36)	156.30 (4.29)	166.76 (16.02)	0.49	175.66 (6.38)	170.38 (22.06)	0.82
TCHOL (mg/dl)	199.65 (1.10)	200.54 (1.54)	166.41 (5.45)	<0.001	200.68 (1.44)	182.89 (11.16)	0.12
HDL (mg/dl)	53.18 (0.45)	54.13 (0.58)	47.63 (2.03)	0.003	52.10 (0.62)	49.69 (3.29)	0.48
HbA1c (%)	5.78 (0.03)	5.74 (0.03)	6.02 (0.12)	0.03	5.83 (0.04)	5.99 (0.11)	0.17
Education				0.9			0.53
Less than 9th grade	328 (10.11%)	183 (5.48%)	8 (6.39%)		134 (5.98%)	3 (2.76%)	
9–11th grade	510 (15.72%)	296 (11.88%)	8 (10.64%)		202 (13.73%)	4 (9.79%)	
High school graduate	811 (24.99%)	472 (25.10%)	14 (21.75%)		312 (23.95%)	13 (22.94%)	
Some college graduate	908 (27.98%)	521 (29.43%)	17 (34.91%)		360 (32.47%)	10 (28.02%)	
College graduate or above	688 (21.2%)	412 (28.11%)	16 (26.30%)		244 (23.88%)	16 (36.50%)	
Marital				0.02			0.78
Never married	345 (10.63%)	206 (10.43%)	4 (3.28%)		132 (10.48%)	3 (7.03%)	
Living with partner	2,046 (63.05%)	1,186 (64.45%)	35 (54.93%)		795 (67.03%)	30 (72.55%)	
Widowed/divorced	854 (26.32%)	492 (25.12%)	24 (41.78%)		325 (22.49%)	13 (20.42%)	
Race				<0.001			0.01
Non-Hispanic White people	1,341 (41.33%)	759 (66.23%)	43 (86.28%)		509 (65.70%)	30 (84.30%)	
Non-Hispanic Black people	750 (23.11%)	447 (12.50%)	13 (8.06%)		284 (12.32%)	6 (6.04%)	
Mexican American	517 (15.93%)	317 (9.54%)	2 (1.46%)		195 (9.44%)	3 (2.44%)	
Hispanic American	298 (9.18%)	177 (5.03%)	1 (0.40%)		118 (4.86%)	2 (0.99%)	
Other race	339 (10.45%)	184 (6.70%)	4 (3.80%)		146 (7.68%)	5 (6.24%)	
Sex				0.8			0.01
Female	1,229 (37.87%)	702 (38.71%)	17 (36.33%)		503 (39.00%)	7 (14.57%)	
Male	2,016 (62.13)	1,182 (61.29%)	46 (63.67%)		749 (61.00%)	39 (85.43%)	
Smoke				0.02			0.01
Never	1,533 (47.24%)	911 (47.66%)	19 (21.91%)		581 (44.23%)	22 (51.66%)	
Former	904 (27.86%)	514 (27.80%)	29 (46.36%)		340 (29.17%)	21 (45.16%)	
Now	808 (24.9%)	459 (24.54%)	15 (31.73%)		331 (26.60%)	3 (3.18%)	
Alcohol				0.02			0.07
Never	390 (12.02%)	205 (8.28%)	6 (9.77%)		176 (9.74%)	3 (3.30%)	
Former	631 (19.45%)	358 (16.69%)	16 (24.40%)		248 (17.86%)	9 (18.43%)	
Mild	1,127 (34.73%)	658 (36.08%)	28 (44.24%)		419 (36.97%)	22 (46.50%)	
Moderate	428 (13.19%)	269 (17.32%)	11 (20.50%)		138 (12.16%)	10 (25.21%)	
Heavy	669 (20.62%)	394 (21.63%)	2 (1.09%)		271 (23.28%)	2 (6.56%)	
MI				<0.001			<0.001
No	3,132 (96.52%)	1,844 (98.20%)	34 (56.10%)		1,222 (97.85%)	32 (75.92%)	
Yes	113 (3.48%)	40 (1.80%)	29 (43.90%)		30 (2.15%)	14 (24.08%)	
Chest pain				<0.001			<0.001
No	3,179 (97.97%)	1,859 (99.03%)	52 (81.95%)		1,233 (98.47%)	35 (61.56%)	
Yes	66 (2.03%)	25 (0.97%)	11 (18.05%)		19 (1.53%)	11 (38.44%)	
Hypertension				0.29			0.003
No	1,248 (38.46%)	765 (43.68%)	14 (34.91%)		462 (41.05%)	7 (13.94%)	
Yes	1,997 (61.54%)	1,119 (56.32%)	49 (65.09%)		790 (58.95%)	39 (86.06%)	
Diabetes				0.01			0.01
No	2,273 (70.05%)	1,343 (76.77%)	31 (58.76%)		876 (74.32%)	23 (54.05%)	
Prediabetes	289 (8.91%)	157 (7.73%)	9 (14.13%)		114 (9.21%)	9 (26.66%)	
Yes	683 (21.05%)	384 (15.50)	23 (27.12%)		262 (16.47%)	14 (19.30%)	
Hyperlipidemia				<0.001			0.004
No	769 (23.7%)	444 (23.22%)	4 (4.01%)		319 (26.10%)	2 (4.71%)	
Yes	2,476 (76.3%)	1,440 (76.78%)	59 (95.99%)		933 (73.90%)	44 (95.29%)	
Take drug for hypertension				<0.001			<0.001
No	1,306 (40.25%)	773 (37.88%)	2 (3.97%)		530 (39.99%)	1 (1.51%)	
Yes	267 (8.23%)	382 (19.42%)	50 (85.46%)		116 (8.82%)	5 (7.55%)	
Other	1,672 (51.53%)	729 (42.71%)	11 (10.57%)		606 (51.19%)	40 (90.94%)	
Take drug for diabetes				<0.001			<0.001
No	1,306 (40.25%)	773 (37.88%)	2 (3.97%)		530 (39.99%)	1 (1.51%)	
Yes	410 (12.63%)	241 (9.75%)	14 (19.28%)		144 (8.06%)	11 (16.35%)	
Other	1,529 (47.12%)	870 (52.37%)	47 (76.75%)		578 (51.95%)	34 (82.13%)	
Take drug for antihyperlipidemic				<0.001			<0.001
No	1,306 (40.25%)	773 (37.88%)	2 (3.97%)		530 (39.99%)	1 (1.51%)	
Yes	756 (23.3%)	382 (19.42%)	50 (85.46%)		284 (23.02%)	40 (83.31%)	
Other	1,183 (36.46%)	729 (42.71%)	11 (10.57%)		438 (37.00%)	5 (15.18%)	

UA, uric acid; PIR, poverty-to-income ratio; WC, waist circumference; BMI, body mass index; MI, myocardial infarction; HDL, high density lipoprotein; TG, triglyceride; BUN, blood urea nitrogen; ALB, albumin; CR, creatinine; TCHOL, Total cholesterol.

### Univariate and multivariate regression analysis

Table 2 presents the outcomes of employing logistic regression analysis to identify the factors contributing to CHD risk. Univariate regression analysis demonstrated that age, race, smoking status, myocardial infarction status, hypertension status, chest pain status, hyperlipidemia status, diabetes mellitus status, waist circumference, UA, CR, BUN, ALB, TCHOL, HDL, HbA1c, and the use of antihyperglycemic/antihypertensive/lipid-lowering medication had a notable association between the emergence of CHD risk in individuals with periodontitis (*P* < 0.05). The variables with a significance level of *P* < 0.05 in the univariate regression analysis were included in the multivariate regression analysis. This analysis revealed that age, race, myocardial infarction status, chest pain status, lipid-lowering medication use, UA levels, and CR levels emerged as the final predictors utilized for constructing the model assessing coronary heart disease risk among patients with periodontitis(*P* < 0.05).

**Table 2:** Weighted univariate and multivariate regression analysis.

Variables	Univariate OR (95% CI)	*P*-value	Multivariate OR (95% CI)	*P*-value
Age (years)	1.09 (1.07, 1.11)	<0.001	1.06 (1.03, 1.10)	0.001
BMI (kg/m^2^)	1.03 (0.99, 1.07)	0.11	/	/
WC (cm)	1.03 (1.01, 1.04)	0.001	/	/
PIR	1.06 (0.91, 1.22)	0.45	/	/
Sleep (hours)	1.03 (0.88, 1.21)	0.68	/	/
Sedentary (min)	1.00 (1.00, 1.00)	0.18	/	/
Exercise (min)	1.00 (1.00, 1.00)	0.17	/	/
UA (mg/dl)	1.38 (1.14, 1.69)	0.002	1.40 (1.06, 1.85)	0.019
CR (mg/dl)	2.34 (1.29, 4.24)	0.01	2.29 (1.41, 3.72)	0.002
BUN (mg/dl)	1.07 (1.05, 1.10)	<0.001	/	/
ALB (mg/dl)	0.40 (0.15, 1.02)	0.05	/	/
TG (mg/dl)	1.00 (1.00, 1.00)	0.58	/	/
TCHOL (mg/dl)	0.98 (0.97, 0.99)	0.001	/	/
HDL (mg/dl)	0.98 (0.96, 1.00)	0.03	/	/
HbA1c (%)	1.18 (1.07, 1.29)	0.001	/	/
Education				
Less than 9th grade	Ref.	Ref.	Ref.	Ref.
9–11th grade	0.96 (0.43, 2.17)	0.93	/	/
High school graduate	1.07 (0.53,2.16)	0.85	/	/
Some college graduate	1.23 (0.52, 2.92)	0.63	/	/
College graduate or above	1.38 (0.69, 2.73)	0.35	/	/
Marital				
Never married	Ref.	Ref.	Ref.	Ref.
Living with partner	2.03 (0.56, 7.33)	0.27	/	/
Widowed/divorced	2.85 (0.91, 8.86)	0.07	/	/
Race				
Non-Hispanic White people	Ref.	Ref.	Ref.	Ref.
Non-Hispanic Black people	0.45 (0.27, 0.74)	0.002	/	/
Mexican American	0.15 (0.06, 0.38)	<0.001	/	/
Hispanic American	0.10 (0.03, 0.33)	<0.001	0.09 (0.01, 0.66)	0.02
Other race	0.53 (0.27, 1.06)	0.07	/	/
Sex				
Female	Ref.	Ref.	Ref.	Ref.
Male	1.74 (0.88, 3.42)	0.11	/	/
Smoke				
Never	Ref.	Ref.	Ref.	Ref.
Former	2.14 (1.31, 3.50)	0.003	/	/
Now	1.00 (0.43, 2.35)	1.00	/	/
Alcohol				
Never	Ref.	Ref.	Ref.	Ref.
Former	1.62 (0.66, 3.97)	0.28	/	/
Mild	1.59 (0.63, 3.96)	0.32	/	/
Moderate	1.88 (0.72, 4.88)	0.19	/	/
Heavy	0.20 (0.04, 1.09)	0.06	/	/
MI				
No	Ref.	Ref.	Ref.	Ref.
Yes	27.56 (13.97,54.36)	<0.001	7.18 (3.20, 16.14)	<0.001
CP				
No	Ref.	Ref.	Ref.	Ref.
Yes	30.82 (14.84, 64.02)	<0.001	15.77 (4.71, 52.77)	<0.001
Hypertension				
No	Ref.	Ref.	Ref.	Ref.
Yes	2.15 (1.28, 3.61)	0.005	/	/
Hyperlipidemia				
No	Ref.	Ref.	Ref.	Ref.
Yes	7.12 (3.06, 16.55)	<0.001	/	/
DM				
No	Ref.	Ref.	Ref.	Ref.
Prediabetes	3.17 (1.57, 6.40)	0.002	/	/
Yes	1.99 (1.15, 3.47)	0.02	/	/
Take drug for hypertension				
No	Ref.	Ref.	Ref.	Ref.
Yes	17.40 (3.75, 80.77)	<0.001	/	/
Other	21.78 (5.49, 86.38)	<0.001	/	/
Take drug for diabetes				
No	Ref.	Ref.	Ref.	Ref.
Yes	26.47 (5.11, 137.04)	<0.001	/	/
Other	20.29 (5.37, 76.69)	<0.001	/	/
Take drug for antihyperlipidemic				
No	Ref.	Ref.	Ref.	Ref.
Yes	54.39 (12.99, 227.83)	<0.001	5.06 (2.03, 12.59)	0.001
Other	4.16 (1.34, 12.99)	0.02	/	/

BMI, body mass index; PIR, poverty-to-income ratio; WC, waist circumference; MI, myocardial infarction; HDL, high density lipoprotein; TG, triglyceride; UA, uric acid; BUN, blood urea nitrogen; ALB, albumin; CR, creatinine; TCHOL, Total cholesterol.

### Development and validation of five machine learning models

**Figure 2:**
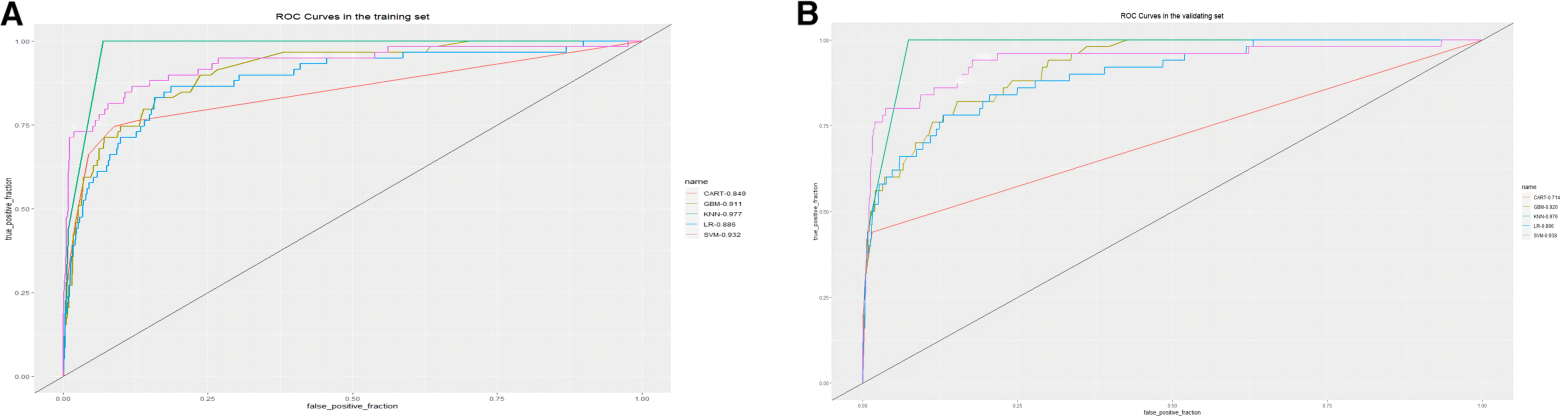
(**A**) ROC curve analysis of 5 ML algorithms in the training set. (**B**) ROC curve analysis of 5 ML algorithms in the validating set.

**Figure 3:**
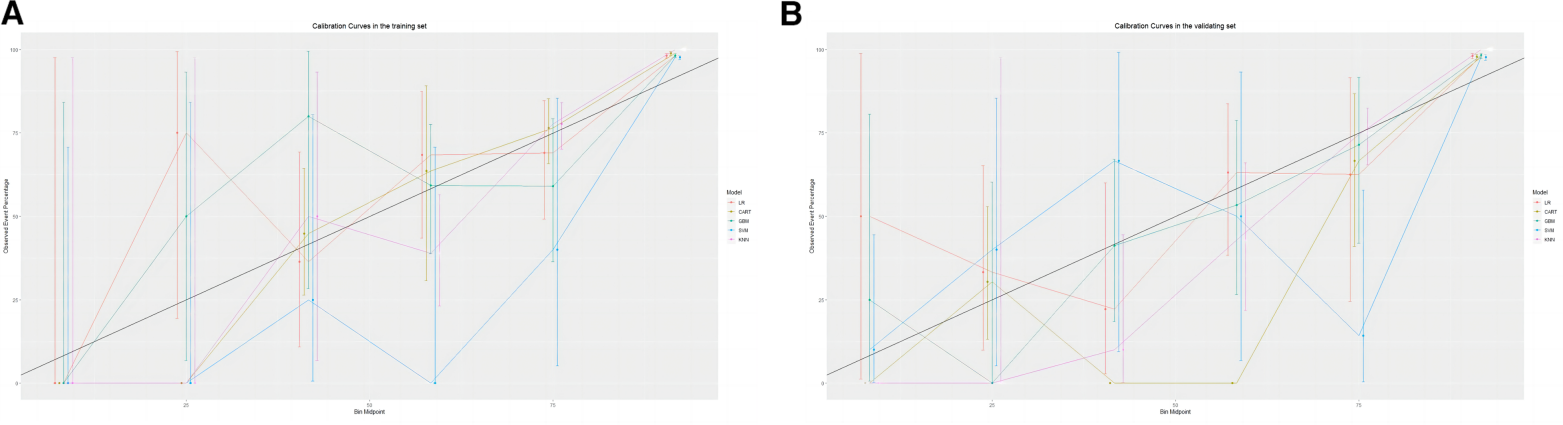
(**A**) Calibration curve analysis of 5 ML algorithms in the training set. (**B**) Calibration curve analysis of 5 ML algorithms in the validating set.

**Figure 4:**
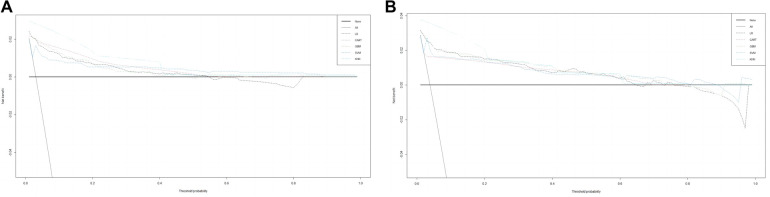
(**A**) DCA curve analysis of 5 ML algorithms in the training set. (**B**) DCA curve analysis of 5 ML algorithms in the validating set.

The training set incorporates five machine learning algorithms, namely LR, CART, GBM, SVM, and KNN, to construct the prediction model. Subsequently, the predictive performance of these models is assessed through ROC curve analysis (Figure 2A). The K-nearest neighbor algorithm model demonstrated the highest predictive performance for assessing the risk of coronary heart disease in the periodontitis population (AUC = 0.977), followed by the support vector machine model (AUC = 0.932), gradient boosting machine model (AUC = 0.911), logistic regression model (AUC = 0.886), and classification and regression tree model (AUC = 0.849). As depicted in Figure 2B, among the five machine learning models in the validation set, the k-nearest neighbor model exhibits superior performance in ROC curve analysis with an AUC value of 0.938. Meanwhile, the accuracy of the prediction results relative to the actual occurrence of events was evaluated by the calibration curves of the training set and validation set. The calibration curve for the training set show that the predictive ability of the k-nearest neighbor model is very similar to the actual results (Figure 3A). Of course, the calibration curve for the validation set show that the k-nearest neighbor model also performs well (Figure 3B). Additionally, the discriminative power of the model was assessed by calculating the Brier score in both the training and validation sets. Amongst the five machine learning models, the k-nearest neighbor algorithm exhibited a superior discrimination with a Brier score of 0.019 in the training set, compared to 0.024 for support vector machine model, 0.024 for gradient boosting machine model, 0.022 for classification and regression tree model, and 0.024 for logistic regression model respectively. Consequently, it can be concluded that the k-nearest neighbor algorithm demonstrates optimal discrimination ability. Furthermore, in the validation set, this algorithm also outperformed others with a Brier score of 0.022 as shown in Table 3. The DCA of the training set demonstrates that among the five machine learning models, the k-nearest neighbor model exhibits superior performance, thereby confirming its qualified clinical utility (Figure 4A). Furthermore, the DCA of the validation set reveals a significant positive net benefit in predicting risk associated with the implementation of the k-nearest neighbor model (Figure 4B). Therefore, the KNN model is selected as the ultimate prediction model.

**Table 3:** Brier scores for training set and validating set.

	Brier score for training set	Brier score for validating set
KNN	0.019	0.022
CART	0.022	0.028
GBM	0.024	0.026
SVM	0.024	0.028
LR	0.024	0.026

### Development of nomogram

**Figure 5:**
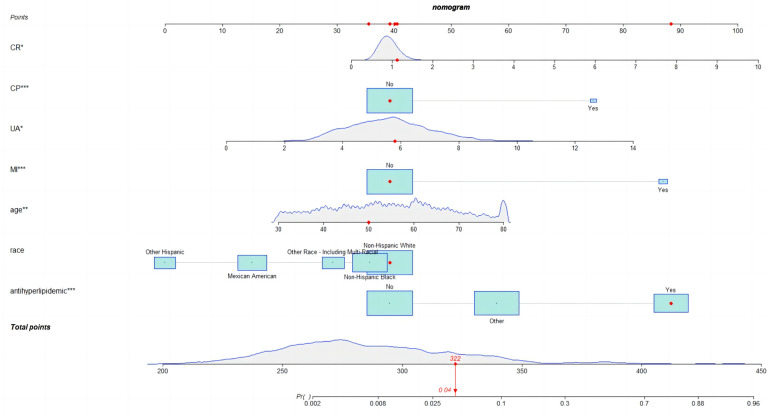
Nomogram for the risk of coronary heart disease for patients with periodontitis.

Given the satisfactory performance of logistic regression predictions, we constructed a nomogram (Figure 5) on the training set to show the practicality and visualization of our model for predicting CHD risk in individuals with periodontitis.

### Relative significance of factors in machine learning algorithms

**Figure 6:**
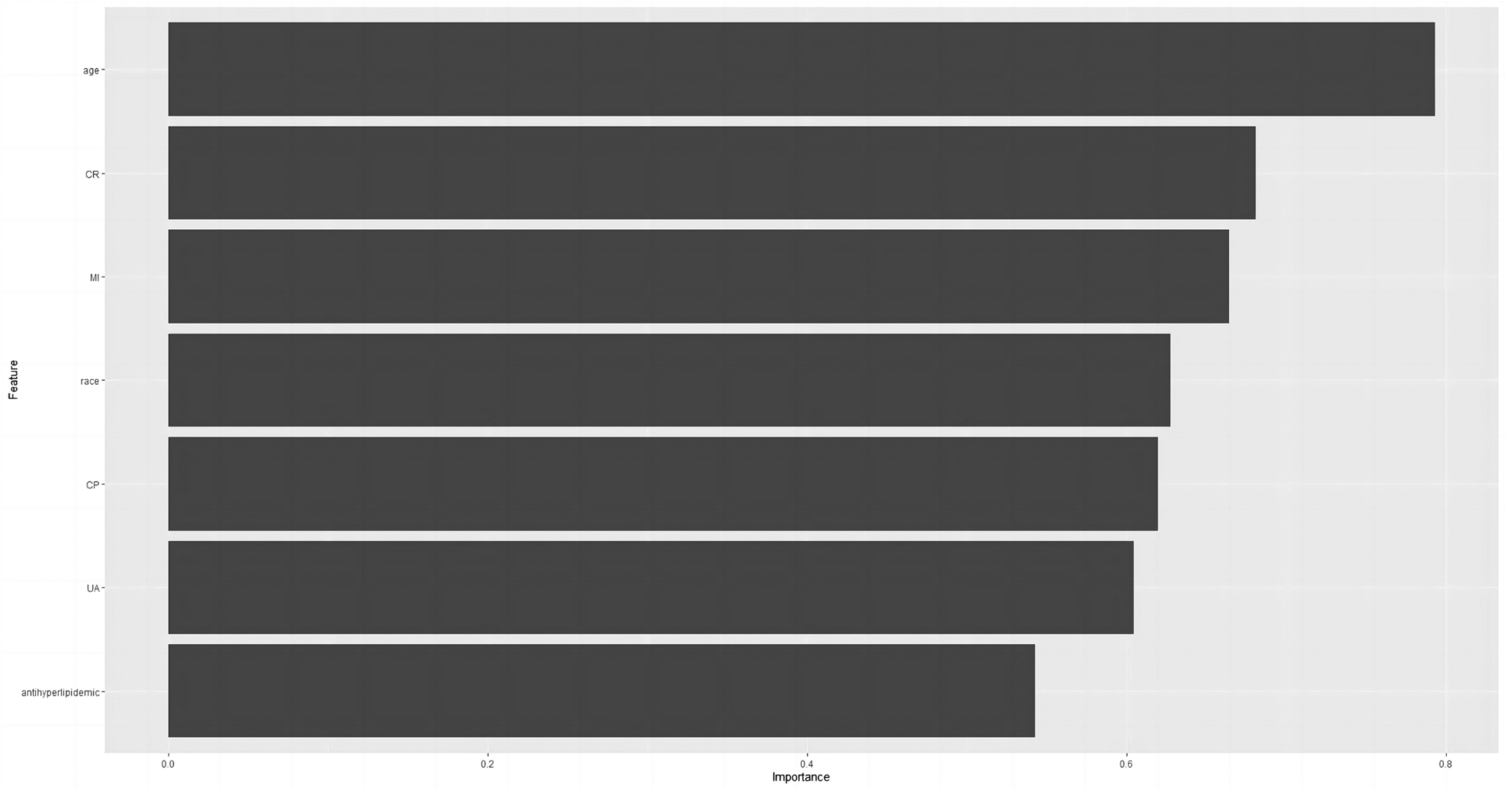
Importance ranking of variables in KNN model.

Based on the final results, we have identified the KNN model as the ultimate predictive model. The relative importance of the variables in the five machine algorithm models is shown in Figure 6. The predictor variables in the KNN model were ranked in descending order of importance, age, CR, myocardial infarction status, race, chest pain status, UA, and lipid-lowering medication use.

## Discussion

By analyzing a total of 3,245 NHANES 2009–2014 participants, we developed and validated five distinct machine learning algorithms (LR, CART, GBM, SVM, and KNN) to accurately predict the risk of CHD in individuals suffering from. The NHANES study used a large stratified, multi-stage sampling design, which weighted the data to more accurately reflect overall population characteristics than unweighted results. Through weighted logistic regression analysis, we identified seven variables: age, race, myocardial infarction status, chest pain status, usage of lipid-lowering medication, UA levels, and CR levels. Notably, each machine learning model exhibited distinct characteristics in terms of identification accuracy, calibration performance, and clinical utility; among them all, the KNN model demonstrated superior predictive ability. Consequently, this machine learning-based approach holds promise for clinicians to estimate disease prevalence within specific populations.

The age factor emerged as the foremost risk determinant in our investigation. Significantly, the mean age of patients diagnosed with periodontitis and concurrent coronary heart disease exceeded that of patients without coronary heart disease by more than a decade. In physiology, aging is an irreversible process characterized by a gradual decline in physiological functions (31). The risk of CHD escalates significantly with advancing age (32). Therefore, the early diagnosis and treatment of chronic diseases such as CHD are crucial, necessitating the development of strategies to prevent coronary heart disease at an early stage and enhance the quality of life among middle-aged and elderly individuals.

Despite notable advancements in reducing the burden of cardiovascular disease within the general population of the United States, persistent racial and ethnic disparities in cardiovascular disease mortality remain evident. Specifically, individuals of black ethnicity in the United States continue to exhibit a heightened susceptibility to cardiovascular disease compared to other racial and ethnic groups (33). The genetic basis may underlie the observed racial disparities. Further research is necessary to comprehensively understand the heterogeneous distribution of cardiovascular disease based on race and ethnicity, as well as to elucidate the underlying factors contributing to racial and ethnic disparities.

Numerous studies have consistently demonstrated a robust correlation among elevated levels of UA and the pathogenesis and progression of coronary atherosclerosis, along with the severity of CHD, cardiovascular mortality, and all-cause mortality (34, 35). Furthermore, logistic regression analysis in our study revealed a significant association between uric acid levels and the risk of CHD. This relationship may be attributed to the induction of oxidative stress, endothelial dysfunction and inflammatory mechanisms triggered by elevated uric acid concentrations, thereby increasing the susceptibility to coronary heart disease (36, 37). Therefore, active uric acid-lowering therapy is imperative in the presence of hyperuricemia concomitant with cardiovascular disorders such as coronary heart disease, aiming to retard the progression of cardiovascular disease and optimize patient prognosis.

The occurrence of myocardial infarction serves as the principal manifestation of coronary artery disease, representing a severe and critical condition (38). Immediate surgical intervention is typically necessary for the management of acute myocardial infarction (39). Chest pain serves as a clinical manifestation of CHD and frequently acts as a precursor to acute myocardial infarction (40).

According to our findings, the positive association between creatinine and the risk of CHD remained robust even after controlling for all potential confounding factors. Previous studies have demonstrated a positive correlation between elevated serum creatinine levels and an augmented risk of cardiovascular diseases, including coronary heart disease (41, 42). The presence of elevated serum creatinine levels typically indicates renal impairment, which is commonly associated with an augmented cardiovascular risk (43). Therefore, it is imperative to reduce serum creatinine levels in order to achieve optimal cardiovascular risk management in patients diagnosed with coronary heart disease.

Our study employed machine learning algorithms to specifically predict the risk of coronary heart disease in individuals with periodontitis, a factor that has been rarely explored in previous research endeavors, despite the extensive evaluation of CHD risk within the general population. To the best of our knowledge, this study presents the pioneering application of a machine learning-based predictive model to evaluate the risk of CHD in participants diagnosed with periodontitis.

Naturally, our study is subject to certain limitations. Firstly, NHANES is based on cross-sectional features of the survey, which makes it difficult to determine causality for the diseases under discussion because of the unclear sequence of events. Secondly, owing to inherent limitations in the NHANES study design, this study was unable to provide prognostic insights into the timing and severity of coronary heart disease. Thirdly, although we partitioned the NHANES dataset into a training set and a validation set in a 6:4 ratio, we did not incorporate external data for assessing the predictive model's validity. Additionally, our chosen population solely consisted of adult individuals residing in the United States, thereby limiting its direct applicability to populations in other countries. Consequently, there is an imperative need for conducting multi-center studies across diverse nations. Lastly, the data we used were all from the NHANES database, including home interviews and mobile examination center (MEC) health checks. This may cause some interference with the accuracy of our data and affect the objectivity of the results.

## Conclusion

In this study, we developed a machine learning-based prediction model to assess the risk of coronary heart disease in patients with periodontitis. Our findings demonstrate that among five machine learning models, the KNN model exhibited superior predictive performance. The implementation of our prediction model enables healthcare professionals to provide early and personalized diagnosis and treatment plans for patients with periodontitis, thereby facilitating effective management of coronary heart disease risk.

## Data Availability

The raw data supporting the conclusions of this article will be made available by the authors, without undue reservation.
